# Genome-Wide Characterization and Gene Expression Analysis of TRP Channel Superfamily Genes in the Migratory Locust, *Locusta migratoria*

**DOI:** 10.3390/genes14071427

**Published:** 2023-07-11

**Authors:** Yong Yang, Wenhui Guo, Mingjun Wang, Daochuan Zhang

**Affiliations:** The International Centre for Precision Environmental Health and Governance, The Key Laboratory of Zoological Systematics and Application, College of Life Sciences, Hebei University, Baoding 071002, China; yongy1217@163.com (Y.Y.);

**Keywords:** TRP superfamily, gene expression, *Locusta migratoria*

## Abstract

The TRP channel superfamily was widely found in multiple species. They were involved in many extrasensory perceptions and were important for adapting to the environment. The migratory locust was one of the worldwide agricultural pests due to huge damage. In this study, we identified 13 TRP superfamily genes in the locust genome. The number of LmTRP superfamily genes was consistent with most insects. The phylogenetic tree showed that LmTRP superfamily genes could be divided into seven subfamilies. The conserved motifs and domains analysis documented that LmTRP superfamily genes contained unique characteristics of the TRP superfamily. The expression profiles in different organs identified LmTRP superfamily genes in the head and antennae, which were involved in sensory function. The expression pattern of different life phases also demonstrated that LmTRP superfamily genes were mainly expressed in third-instar nymphs and male adults. Our findings could contribute to a better understanding of the TRP channel superfamily gene and provide potential targets for insect control.

## 1. Introduction

Transient receptor potential (TRP) channels are members of cation channels, which are evolutionarily conserved proteins that have been widely identified in many model and non-model species, such as *Drosophila melanogaster* [1], *Tribolium castaneum* [2], mice [3], humans [4], *Spodoptera littoralis* [5], *Lygus hesperus* [6], and *Nilaparvata lugens* [7]. However, they are not present in plants except for algae. TRP channel proteins play a role in a variety of extrasensory perceptions, such as thermosensation, olfaction, mechanosensation, and vision [1,8]. Members perform both similar and different functions. Members of the TRP channel family, typically characterized by six conserved transmembrane (TM) domains with a pore loop between TM 5 and 6, are in the cell membrane [9]. Based on the homology of the primary amino acid sequence, TRPs are classified into seven subfamilies, including TRPA (ankyrin) [10], TRPC (canonical), TRPM (melastatin), TRPML (mucolipin), TRPN (nompC), TRPP (polycystic), and TRPV (vanilloid) [8]. They are also broadly divided into two groups based on various subfamily sequences. One group is homologous to *Dmtrp*, including TRPA, TRPV, TRPC, TRPM, and TRPN. The other group of TRP channels consists of the TRPP and TRPML subfamilies, which lack the multiple ankyrin repeats and TRP domains characteristic of one-group channels [11]. The various subfamilies contain one or more individuals. For instance, the TRPA subfamily has four members in *Drosophila*, named TRPA1, Painless (Pain), Pyrexia (Pyx), and Water witch (Wtrw) [12]. The TRPC subfamily has three members, including canonical TRP, TRPL (TRP-like), and TRPγ (TRPgamma). The TRPV subfamily consists of Inactive (Iav) and Nan-chung (Nan) [13]. Meantime, the TRPP subfamily also includes two members as polycystin-2 (Pkd2) and the atypical *Drosophila* brivido proteins (Brv) [14]. In contrast, the TRPM, TRPN, and TRPML subfamilies are limited to single members. TRP channels have evolutionary differences among different species. Both the TRPA and TRPP subfamilies are distinct in terms of their numbers. There are five, four, four, and two TRPA members in *T. castaneum*, *Pediculus humannus, Acyrthosiphon pisum* and *Caenorhabditis elegans*, respectively [15,16,17,18,19]. Some insects lack the TRPP subfamily, such as *Apis mellifera, Solenopsis invicta*, *Bombyx mori*, and *Nasonia vitripennis* [17].

Previous studies have shown that TRP channel superfamily genes have a crucial role in insect growth and development. TRP channels were first discovered in *dmtrp* mutants that responded to light [20]. Despite responding to light as well, TRPL can regulate female egg laying in *N. lugens* and *D. melanogaster* [21]. *Sl*TRPγ has been identified in both pupal and adult male antennae and is essential for insect olfactory in the transduction of *S. littoralis* [5]. *Rpro*Iav is related to thermal sensitivity [22]. Additionally, *Dm*Nan-Iav heteromers are silenced under insecticide and prevent insect feeding [23]. However, recent studies have found that *Dm*Nan and *Dm*Wtrw can also form heteromers and be activated by insecticides. Meanwhile, Wtrw increases the binding affinity of Nan for binding afidopyropen [24]. The NompC channel, which belongs to the TRPN subfamily, has the largest number of ankyrin repeats [9]. NompC is known as the touch sensation and is essential for exploring the environment. DmNompC is highly expressed in class III neurons and activated by mechanical force [25,26]. In *Drosophila*, NompC mutant adults are severely uncoordinated, and mutant larvae display severe defects in behavioral responses to gentle touches [26]. Loss of NompC abolishes active amplification in *Drosophila* antennae [27]. NlNompC is also important for light touch sensation. The survival rate and touch score of the third-instar nymphs are lower after injecting ds*NlNompC* [28]. Subsequently, NompC loss of function mutants lack sensitive hearing, which indicates that NompC is an important component of hearing [29].

Thermotaxis and temperature acclimation are connected to three TRPA members, including TRPA1, Painless, and Pyx in *T. castaneum* [30]. Since insects are ectothermic creatures, their body temperatures can fluctuate depending on the surrounding temperature. Temperature can also act as a rhythm to influence the physiological behavior of insects. DmPyx can enhance the tolerance to high temperatures. BmTRPA1 can regulate diapause behavior due to temperature [31]. However, different insects have their own suitable temperatures for survival. When the ambient temperature is out of range, it will cause insects to avoid the discomfort caused by temperature. Four TRPA variants (AlucTRPA1A-D) cloned were activated by various temperatures in *Apolygus lucorum* [32]. A recent study also has found that white-backed planthoppers prefer a cold region by TRPA1 rather than their comfort temperature zone [33].

Chemical sensations can be divided into olfactory and gustatory and are important in feeding and reproduction. These chemicals are sensed by olfactory and gustatory receptors on specific organs of insects, including the head, leg, wing, and antennae. Although TRP channel genes do not directly sense chemicals, they can participate in the transmission of signals from these chemical substances. The *osm-9* mutant encoding a protein with structural similarity to the TRP channel superfamily gene cannot be affected by odors in *C. elegans* [34]. *AgTRPA1* is expressed in the antennae and can be activated by citronella in *Anopheles gambiae* [35]. In mammals, citronella also inhibits the expression of TRPM2 and lowers oxidative stress-induced mitochondrial damage [36].

The migratory locust *L. migratoria* is one of the most harmful pests to worldwide agricultural production. Locusts can be either solitarious or gregarious depending on population density [37]. Phenotypic plasticity can be adapted to environmental changes. At present, olfactory receptors and the regulatory function of endocrine hormones are currently the main topics of research on migratory locusts [38,39]. Research has found that LmOR17 and LmOR21 can sense E-2-hexenal and hexanal to decrease the vomiting intensity [40]. 4-Vinyanisole is an aggregation pheromone and promotes the synchrony of sexual maturation by LmOR35 [41]. Juvenile hormones, 20-hydroxyecdysone, and insulin can be involved in regulating locust vitellogenesis and are critical for reproduction and growth [42]. Although TRP superfamily genes are involved in a wide range of physiological processes and have been reported in other insects, there are few reports on the regulation of TRP channel superfamily genes in locusts.

In this study, we investigated LmTRP superfamily genes in the *L. migratoria*. The gene structure of subfamily members is displayed. The expression profile of members was detected. Our studies could provide more helpful information for understanding TRP channel superfamily genes and might offer potential targets for insect control.

## 2. Materials and Methods

### 2.1. Insects

The migratory locusts were collected from the laboratory of the College of Life Sciences, Hebei University, and reared on fresh wheat seedlings every day. All eggs, nymphs, and adult locusts grew at a greenhouse under a 14 h light and 10 h dark photoperiod at 30 ± 2 °C, 60 ± 10% relative humidity.

### 2.2. Gene Identification and Sequence Analysis

TRP superfamily protein sequences of *D. melanogaster*, *B. mori*, and *N. lugens* were downloaded from the NCBI database (National Center for Biotechnology Information (nih.gov) (accessed on 17 April 2023)). All accession numbers of the TRP superfamily in this study are listed in Table S2. TRP superfamily protein sequences of *L. migratoria* were obtained using BLASTP with an e-value < 10^−5^ and a similarity of than 30% in *L. migratoria* genomic website (LocustBase-Locust Genome data (accessed on 17 April 2023)) [38]. The conserved domains were predicted in NCBI CDD (Conserved Domains Database (CDD) and Resources (nih.gov) (accessed on 17 April 2023)). The conserved motifs were predicted in the MEME Suite (MEME—MEME Suite (meme-suite.org) (accessed on 19 April 2023)). TRP superfamily protein sequences from different species were aligned by MUSCLE Wrapper and trimmed by trimAl Wrapper in TBtools. The phylogenetic tree was constructed with the neighbor-joining (NJ) method in MEGA11 [43]. A bootstrap procedure evaluated the reliability of the phylogenetic tree with 1000 replications. The conserved domains, motifs, and gene locations were displayed by using TBtools [44].

### 2.3. Sample Preparation

During the feeding process, to examine the expression levels of different developmental stages, samples are randomly collected first-instar nymph, the second-instar nymph, the third-instar nymph, the four-instar nymph, the fifth-instar nymph, and male and female adults, which ranged from after 1 day to 9 days post adult eclosion. The head, wings, and antenna of the four-instar nymph locusts were collected to RNA-Seq. All samples had three biological replicates, and each replicate had more than three locusts.

### 2.4. Total RNA Extraction and cDNA Synthesis

Total sample RNAs were isolated from different developmental stages and adult tissues using RNA-easy^TM^ Isolation Reagent (Vazyme, Nanjing, China, Cat. R701-01) following the manufacturer’s protocol. All experimental steps were performed at low temperatures or on ice. The concentration of the extracted RNA was determined using a NanoDrop 2000 spectrophotometer (Thermo Fisher Scientific, Waltham, MA, USA). The RNA integrity was evaluated following 1.5% agarose gel electrophoresis. The 1 μg RNA was used to synthesize first-stand cDNA using HiScript^®^ III All-in-one RT SuperMix Perfect for qPCR (Vazyme, Nanjing, China, Cat. R333-01) following the manufacturer’s recommendations. The cDNA product was diluted 10 times. All samples were stored at −80 °C in order to further utilize for qRT-PCR.

### 2.5. RNA-Seq and Differential Expression Analysis

Total RNA of each sample was extracted using TRIzol Reagent (Invitrogen, Carlsbad, CA, USA)/RNeasy Mini Kit (Qiagen, Milan, Italy)/RNA-easy^TM^ Isolation Reagent (Vazyme, Nanjing, China). Total RNA of each sample was quantified and qualified by Agilent 2100 Bioanalyzer (Agilent Technologies, Palo Alto, CA, USA), NanoDrop (Thermo Fisher Scientific Inc, Carlsbad, CA, USA), and 1% agarose gel. A total of 1 μg RNA with a RIN value above 7 was used for the following library preparation. Next-generation sequencing library preparations were constructed according to the manufacturer’s protocol (NEBNext^®^ Ultra™ RNA Library Prep Kit for Illumina^®^). The poly(A) mRNA isolation was performed using NEBNext Poly(A) mRNA Magnetic Isolation Module (NEB, Ipswich, UK) or Ribo-Zero™ rRNA Removal Kit (Illumina, San Diego, CA, USA). The mRNA fragmentation and priming were performed using NEBNext First-Strand Synthesis Reaction Buffer and NEBNext Random Primers. First-strand cDNA was synthesized using ProtoScript II Reverse Transcriptase, and the second-strand cDNA was synthesized using Second AxyPrep vStrand Synthesis Enzyme Mix. The purified double-stranded cDNA by AxyPrep Mag PCR Clean-up (Axygen, Wujiang, China) was then treated with End Prep Enzyme Mix to repair both ends and add a dA-tailing in one reaction, followed by a T-A ligation to add adaptors to both ends. Size selection of Adaptor-ligated DNA was then performed using AxyPrep Mag PCR Clean-up (Axygen, Wujiang, China), and fragments of ~360 bp (with the approximate insert size of 300 bp) were recovered. Each sample was then amplified by PCR for 11 cycles using P5 and P7 primers, with both primers carrying sequences that can anneal to flow cells to perform bridge PCR and P7 primer, carrying a six-base index allowing for multiplexing. The PCR products were cleaned up using AxyPrep Mag. Then libraries with different indices were multiplexed and loaded on an Illumina HiSeq instrument according to the manufacturer’s instructions (Illumina, San Diego, CA, USA). Sequencing was carried out using a 2× 150 bp paired-end [16] configuration; image analysis and base calling were conducted by the HiSeq Control Software (HCS) + OLB + GAPipeline-1.6 (Illumina) on the HiSeq Instrument. Transcriptome data had been submitted to NCBI. (GenBank BioProject ID: PRJNA974177). Br-CA transcriptome data were downloaded from the Genome Sequence Archive in National Genomics Data Center under accession number CRA003038 [41]. The raw data were processed by FastQC and Cutadapt. Clean reads were mapped to locust genome sequence by using HISAT2. The read number of all genes was quantified by using featureCounts. The heatmap of LmTRP superfamily genes was constructed using the value of row-scaled counts with the heatmap3 package (version 1.1.9) in R (version 4.2.1). DEGs were performed by using the DESeq2 package (version 1.36.0) with |log2Foldchange| > 1 and significance levels at *p*-adjust < 0.05.

### 2.6. Quantitative Real-Time Polymerase Chain Reaction (qRT-PCR)

RT-qPCR assays were conducted with SYBR qPCR Master Mix (Vazyme, Nanjing, China, Cat. Q711-02) by using Bio-RAD CFX Connect Real-Time System. The qPCR primers were designed by using Primer 3 (https://primer3.ut.ee/ (accessed on 30 April 2023)), and all primers were listed in Table S3 in this study. The following two-step standard procedure was 95 °C for 3 min, and then 40 cycles at 95 °C for 10 s followed by 57 °C for 30 s, finally, 95 °C for 15 s, 57 °C 60 s and 95 °C 15 s. The CT values were used to calculate the relative expression levels using the 2^−ΔΔCt^ method. *LmGAPDH* was used as internal control. All samples were subjected to three biological replicates.

### 2.7. Statistical Analysis

All qRT-PCR results were analyzed using GraphPad Prism 6.0 in this study. Significant differences among samples were assessed by one-way ANOVA (Tukey’s multiple comparisons test, *p* < 0.05). Data were mean ± SD.

## 3. Results

### 3.1. The Identification of TRP Superfamily Genes of L. migratoria

A total of 13 TRP superfamily genes were identified in the locust genome database by BLASTP. The TRP subfamilies were renamed based on sequence homology with other insect TRP genes (Table 1). And sequence homologies of LmTRP superfamily proteins were more than 30% with *N. lugens* and *D. melanogaster* (Table S1). Among these 13 genes, 6 genes contained complete ORFs, and the other genes only had a partial sequence. The CDS lengths were from 1395 bp to 4587 bp. Thus, the proteins encoding TRP superfamily genes contained 465 to 1529 amino acids. And their molecular weights ranged from 51,742.73 to 174,403.82 daltons, with isoelectric point ranking from 4.98 to 9.22.

### 3.2. The Number of TRP Superfamily Genes in L. migratoria and Other Insects

To explore the number of *L. migratoria* and other insect TRP superfamily genes, some insect TRP genes were obtained from the NCBI website (Table 2), including *Bactrocera dorsalis*, *B. mori*, *A. mellifera*, *D. melanogaster*, and *N. lugens*. The number of TRP genes in these insect species was from 13 to 16. These contain all TRP channels subfamily members in six insects. However, the numbers and members of the TRPA and TRPP subfamilies were different. In contrast, there was no *Brv* gene in *L. migratoria*, *B. mori*, *A. mellifera*, and *N. lugens*.

### 3.3. Phylogenetic Analysis of TRP Superfamily Genes

**Figure 1:**
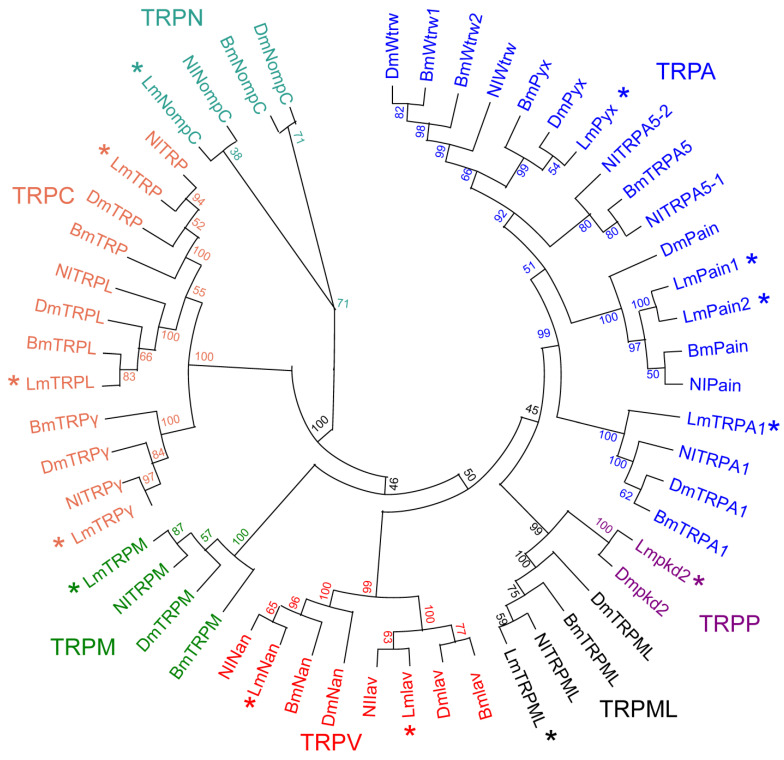
Phylogenetic tree was constructed with the neighbor-joining (NJ) method in MEGA11. A bootstrap procedure evaluated the reliability of the phylogenetic tree with 1000 replications. Phylogenetic tree analysis of TRP superfamily in *L. migratoria* and other insects, including *D. melanogaster*, *N. lugens*, and *B. mori*. Various colors represent different TRP subfamilies. Blue, purple, black, red, green, orange, and cyan represent TRPA, TRPP, TRPML, TRPV, TRPM, TRPC, and TRPN subfamily, respectively. * Represents subfamilies of *L. migratoria* genomic.

To investigate the evolutionary classification of the LmTRP superfamily, a neighbor-joining phylogenetic tree was constructed based on different TRP amino acid sequences from *L. migratoria*, *B. mori*, *D. melanogaster*, and *N. lugens* (Figure 1). According to previous studies and phylogenetic tree analysis, the LmTRP superfamily could be divided into seven subfamilies. The TRPA subfamily had the most members, including LmTRPA1, LmPyx, LmPain1, and LmPain2. TRPP, TRPN, TRPML, and TRPM subfamilies had only one member, respectively. The accession numbers of all TRP superfamily proteins are in Table S2.

### 3.4. Phylogenetic, Conserved Motifs, and Domains in L. migratoria

**Figure 2:**
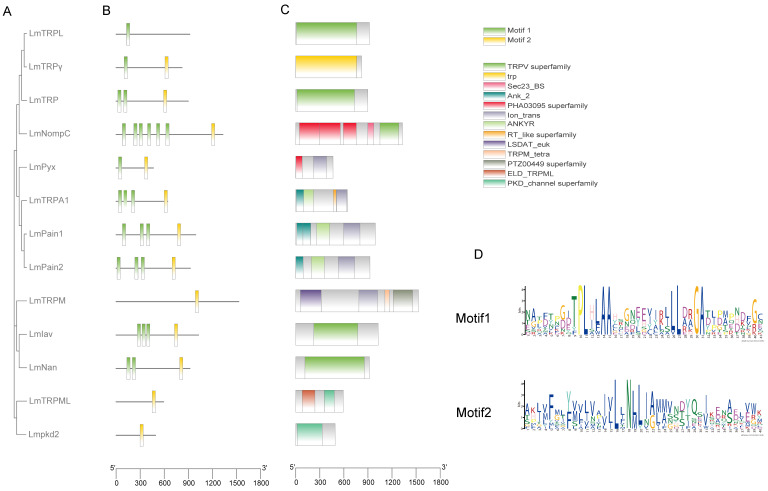
Phylogenetic, conserved domains, structure, and motifs of TRP channel superfamily genes in *L. migratoria*: (**A**) phylogenetic tree, (**B**) conserved motifs, (**C**) conserved domains, (**D**) motif logo.

To analyze the relationship among LmTRP superfamily genes, a phylogenetic tree was produced with amino acid sequences (Figure 2A). LmTRP superfamily genes all contained motif2, apart from LmTRPL. In contrast, motif1 was absent from LmTRPM, LmTRPML, and Lmpkd2. The number of them was also different. LmNompC was the most with five (Figure 2B, D). A TRPV superfamily domain was present in LmIav, LmTRP, LmTRPL, LmNan, and LmNompC. LmPyx, LmPain1, LmPain2, LmTRPA1, and LmTRPM contained an Ion_trans domain. LmPain1, LmPain2, and LmTRPA1 contained Ank_2 and ANKYR domains. LSDAT_euk and PTZ00449 superfamily domains were only contained in LmTRPM. LmTRPγ contained a trp domain. Lmpkd2 and LmTRPML both had a PKD_channel superfamily domain (Figure 2C).

### 3.5. Location of TRP Superfamily Genes in Genome of L. migratoria

**Figure 3:**
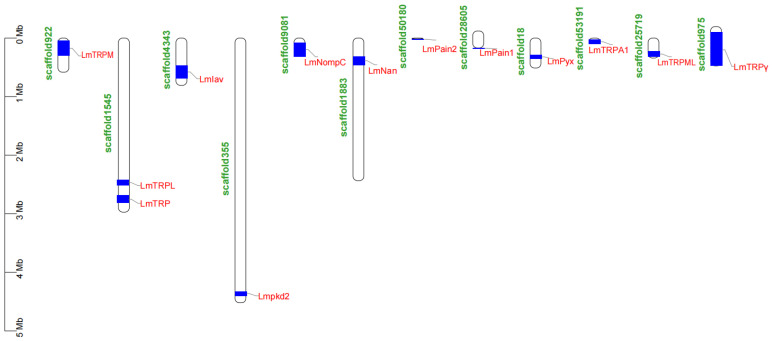
Location of TRP channel superfamily genes in genome of *L. migratoria*.

Based on the *L. migratoria* genome, a gene placement map for the LmTRP superfamily genes was created (Figure 3). There were 13 TRP superfamily genes spread over 12 scaffolds. *LmTRP* and *LmTRPL* were all found in scaffold1545. The scaffolds varied in length. Scaffold355 had the most length; scaffold50180 had the least length. Most of them were occupied by LmTRP genes such as *LmNompC*, *LmPain2*, *LmTRPA1*, and *LmTRPγ*.

### 3.6. Expression Patterns Analysis of TRP Superfamily Genes

**Figure 4:**
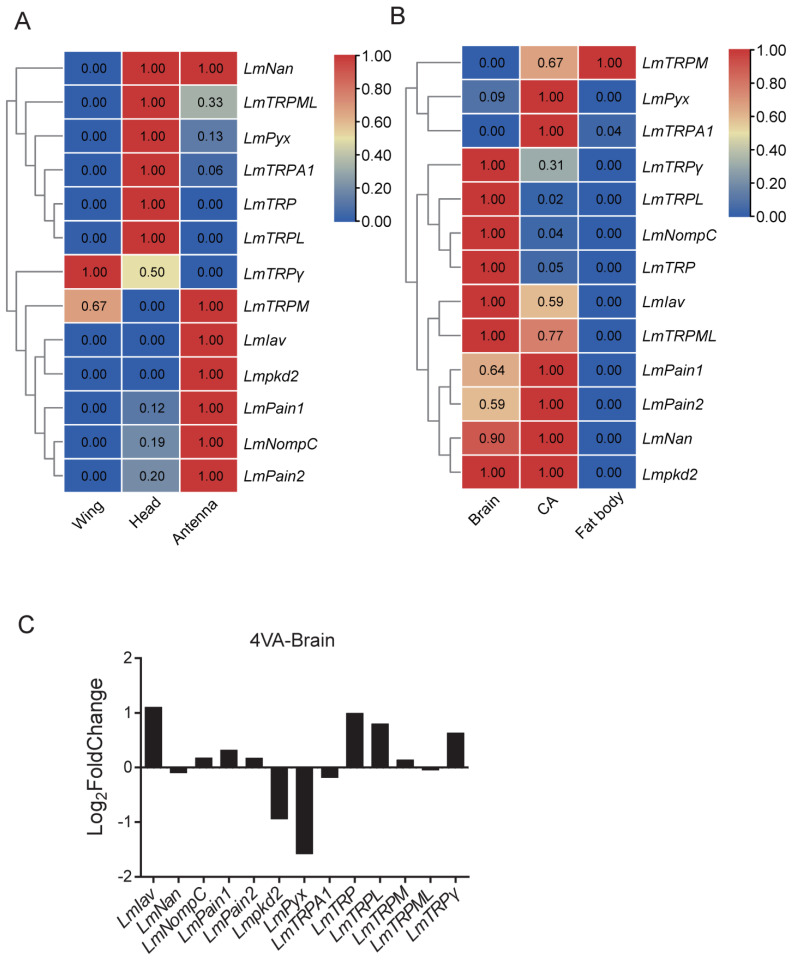
The expression profile of LmTRP superfamily genes in different organs of *L. migratoria.* Transcriptome data were used to investigate the expression levels in (**A**) wings, head, and antennae and (**B**) brain, CA (corpora allatum), and fat body. (**C**) The log_2_Foldchange values of LmTRP superfamily genes were displayed under 4VA conditions.

To understand the expression pattern of LmTRP superfamily genes, RNA-Seq data from different tissues were used. The head, antennae, and wings are important external organs for sensing environmental changes. *LmNan*, *LmTRPML*, *LmPyx*, *LmTRPA1*, *LmTRP*, and *LmTRPL* had higher expression levels in the head compared to wings and antennae. In contrast, the expression levels of *LmTRPM*, *LmIav*, *Lmpkd2*, *LmPain1*, *LmPain2*, and *LmNompC* were the highest in the antennae. *LmTRPγ* was only higher expressed in the wing (Figure 4A). To clarify the expression of the internal tissues on LmTRP superfamily genes, RNA-Seq data were downloaded (accession number CRA003038). Most LmTRP superfamily genes had more expression in the brain and corpus allatum. By contrast, the expression level of *LmTRPM* was highest in the fat body (Figure 4B). 4VA (4-vinylanisole) was a kind of aggregation pheromone [45]. Additionally, it was reported in LmOr35 and could sense 4VA to promote the synchrony of sexual maturation in female locusts [41]. OR could affect the expression of some TRP genes [5]. To explore whether 4VA regulated TRP genes, DEGs were produced under 4VA-treated conditions with fourth-day adults by using DESeq2. The results showed that the expression levels of *LmIav*, *LmTRP*, *LmTRPL*, and *LmTRPγ* increased, and *Lmpkd2* and *LmPyx* decreased (Figure 4C). However, there were no substantial differences between them (*p* > 0.05).

**Figure 5:**
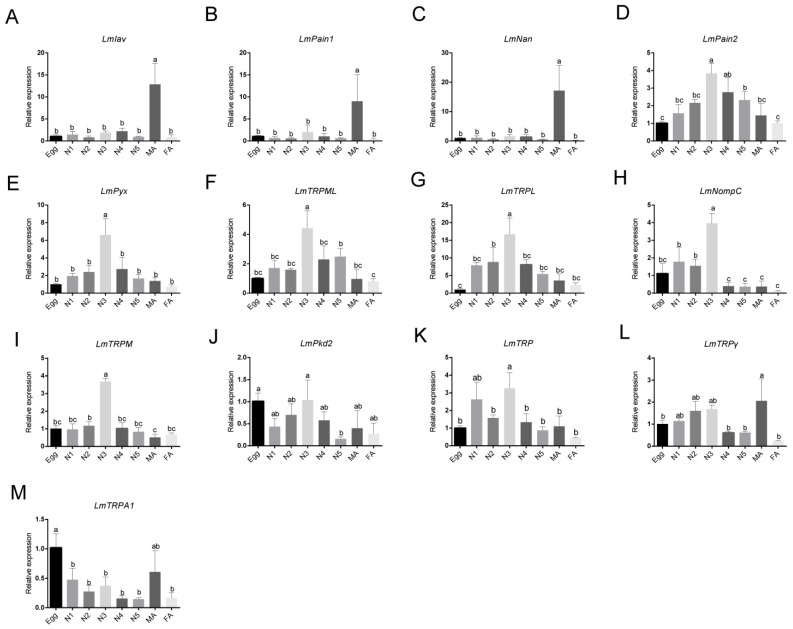
The expression profiles of LmTRP superfamily genes (**A**) *LmIav*, (**B**) *LmPain1*, (**C**) *LmNan*, (**D**) *LmPain2*, (**E**) *LmPyx*, (F) *LmTRPML*, (**G**) *LmTRPL*, (**H**) *LmNopmC*, (**I**) *LmTRPM*, (**J**) *Lmpkd2*, (**K**) *LmTRP*, (**L**) *LmTRPγ*, (**M**) *LmTRPA1* were determined by qRT-PCR assays in various growth and development phases, including eggs, N1 (the first-instar nymph), N2 (the second-instar nymph), N3 (the third-instar nymph), N4 (the fourth-instar nymph), N5 (the fifth-instar nymph), MA (male adult), and FA (female adult). The a, b, and c indicated significant differences (*p* < 0.05). *LmGAPDH* was used as internal control. All samples were subjected to three biological replicates. Data were mean ± SD.

To further determine the expression pattern of LmTRP superfamily genes, qRT-PCR assays were used to identify the expression levels in different growth phases. Multiple phases were tested, including eggs, nymphs, and adults. Results showed that mRNA expression levels of LmTRP superfamily genes were found in all growth phases (Figure 5). *LmNan*, *LmIav*, and *LmPain1* were mainly expressed in male adults (Figure 5A–C). *LmPain2*, *LmPyx*, *LmTRPML*, *LmTRPL*, and *LmNompC* all showed a similar pattern of expression. Their expression levels first increased with age in the first-, second-, and third-instar nymphs. When the fourth-instar nymphs arrived, expression began to decline (Figure 5D–H). The highest expression of *LmTRPM* was detected in the third-instar nymphs (Figure 5I). The lowest expression of *LmPkd2* was found in the fifth-instar nymphs (Figure 5J). *LmTRP* was mainly detected and higher expressed in the first- and third-instar nymphs (Figure 5K). *LmTRPγ* exhibited different expression profiles (Figure 5L). The expression level of *LmTRPA1* was decreasing. Nevertheless, there was an increased expression in male adults (Figure 5M).

## 4. Discussion

TRP channel superfamily genes are involved in sensory signals, cellular activities, and signaling cascades. In this work, we study LmTRP superfamily genes based on sequence homologies by blasting in *L. migratoria* genomic. We discovered 13 LmTRP subfamily genes. The number of TRP superfamily genes has no obvious change compared with other insects (Table 2). The results suggest that TRP channels are evolutionarily conserved proteins. Seven subfamilies of TRP channels are discovered in the locust individuals. However, there are some lost subfamilies in other insects. The TRPP subfamily is absent from reported Hymenoptera insects, including *A. mellifera* and *N. vitripennis* [16]. In contrast, only six subfamilies are found in the Lepidoptera insect *Pieris rapae* [15]. What is more, it has been reported that *B. mori* does not have the TRPP subfamily [17]. The TRPP subfamily has two members, including Pkd2 and Brv. Pkd2 and Brv have been linked to cold stress in fly antennae [46,47]. A recent study has also documented that Brv can sense gentle touch in *Drosophila* [48]. This indicates that TRPP can be replaced by another TRP subfamily or pathway throughout evolution. The number of TRPA subfamily members varies between species. There is only one TRPA member in mammals. Four out of thirteen genes are TRPA members in *L. migratoria*. In contrast, *A. mellifera* and *B. mori* have five and six members. Different numbers prove that the TRPA subfamily of insects expands over the course of evolution. These suggest that gene family members may expand and lose during the evolution of various species.

Two motifs are searched for from all LmTRP superfamily genes, which are the ankyrin repeat and ion transport protein, respectively. TRP channel has six transmembrane domains and a variable number of ankyrin repeats in N-terminal, except for TRPM, TRPP, and TRPML subfamilies [8,49]. As a matter of fact, LmTRPL do not turn out as expected. An incomplete open reading frame may result in the absence of ion transport proteins in LmTRPL. PrNan has four transmembrane domains [15]. NlTRPM and NlTRPML also do not search ankyrin repeat as expected [7]. Locust genome consists of many scaffolds due to large size and sequencing technology. LmTRP superfamily genes are in independent scaffold except for scaffold1545. However, the full length of LmPain2, LmTRPA1, and LmTRPγ might consist of two or more scaffolds. NlTRPγ is in four scaffolds. NlTRPM, NlIav, and NlTRPML are in two scaffolds [7,50].

Insects are ectothermic animals. Changes in the external environment are crucial to the growth and development of insects. Therefore, sensing the external environment has become an important means for insects to adapt for survival. The transcriptome data analysis of different organs approves the highest expression of TRP superfamily genes in the brain, head, and antennae. Antennae are the main chemosensory organ that has a number of ORs (odorant receptors) [40]. Multiple ORs are also expressed on the palps of the head and respond to vomiting by a higher brain area, the accessory calyx [51]. CpTRPA5 belongs to a TRPA subfamily and is expressed in antennae [10]. *SlTRPγ* is identified in the male antennal EST database and is involved in the olfactory system [5]. TRP mutants in *D. melanogaster* are deficient in olfactory adaptation [52]. DmPain also inhibits courtship behavior between males through modulating olfactory sensation [53]. OR35 can sense 4VA to promote sexual maturation in locusts. Expression change might show that TRP superfamily genes are functional under 4VA treatment. *TcTRPA1* can be predominantly expressed in the antenna and induce repellency behavior [54]. *AgTRPA1* is also activated by temperature in the antennae [35]. *DmTRPγ* can regulate food consumption in Drosophila neuroendocrine cells [55]. This suggests that the head and antennae are the main sensory organs and are crucial for adapting to environmental changes.

Insects are exposed to stimuli brought by varied environments throughout their lives. TRP channel genes in different developmental stages of *B. dorsalis* are mainly expressed in adults [18]. Similarly, expression levels of multiple TRP channel genes were lowest in the eggs and relatively higher in adults compared to the nymph stage in brown planthopper [7]. However, there are the same and various expression profiles in locusts. *LmNan*, *LmIav*, *LmPain1*, and *LmTRPγ* have higher expression levels in adults. *LmPain2*, *LmPyx*, *LmTRPM*, *LmTRPL*, *LmTRPML*, and *LmTRP* have higher expression levels in the third-instar nymphs than in adults. The third instar is an important developmental stage in locusts, at which there is a greater mortality rate due to changes in the environment leading to some kind of intestinal disease, although we do not have direct evidence. BmTRPA1 is thermosensitive and affects the determination of diapause in the egg stage [56]. *LmTRPA1* has a higher expression in eggs as well. These indicate that TRPA1 is important for egg development.

## 5. Conclusions

In conclusion, we identified seven subfamilies that contained thirteen TRP genes in *L. migratoria*. Phylogenetic tree analysis compared the relationship between locusts and other insects. Furthermore, conserved functional domains, motifs, and gene location analysis were useful for understanding the functional differences among TRP genes. In addition, we also determined the expression pattern of TRP genes in different growth development and tissues. Additionally, these offer a theoretical scientific foundation for potential biological pest control.

## Figures and Tables

**Table 1:** TRP superfamily genes identified from *L. migratoria*.

Gene Name	Genomic ID	CDS Length (bp)	Amino Acid (aa)	Molecular Weight (Da)	Isoelectric Point
*LmIav*	LOCMI10002	3081	1026	114,537.08	8.81
*LmNan*	LOCMI14722	2757	918	102,814.22	5.9
*LmNompC*	LOCMI07947	3990	1329	146,348.41	9.22
*LmPain1*	LOCMI06538	2976	991	112,666.78	5.41
*LmPain2*	LOCMI03163	2775	924	105,285.22	5.34
*Lmpkd2*	LOCMI15283	1473	490	56,483.04	4.98
*LmPyx*	LOCMI07175	1395	465	51,742.73	9.1
*LmTRPA1*	LOCMI03266	1929	643	72,038.88	8.89
*LmTRP*	LOCMI14741	2697	899	103,911.28	8.42
*LmTRPL*	LOCMI14740	2754	918	105,869.56	6.32
*LmTRPM*	LOCMI11192	4587	1529	174,403.82	5.97
*LmTRPML*	LOCMI07586	1779	592	67,777.94	6.42
*LmTRPγ*	LOCMI11247	2463	821	94,101.82	7.34

**Table 2:** The numbers of TRP superfamily genes in *L. migratoria* and other insects.

Species Name	TRPV		TRPA					TRPC			TRPM	TRPML	TRPN	TRPP		
	Iav	Nan	Pain	Wtrw	TRPA5	Pyx	TRPA1	TRP	TRPL	TRPγ	TRPM	TRPML	NompC	pkd2	Brv	Total
*L. migratoria*	1	1	2	0	0	1	1	1	1	1	1	1	1	1	0	13
*B. dorsalis*	1	1	1	1	0	1	1	1	1	1	1	1	1	2	1	15
*B. mori*	1	1	1	2	1	1	1	1	1	1	1	1	1	0	0	14
*A. mellifera*	1	1	1	1	2	1	0	1	1	1	1	1	1	0	0	13
*D. melanogaster*	1	1	1	1	0	1	1	1	1	1	1	1	1	1	3	16
*N. lugens*	1	1	1	1	2	0	1	1	1	1	1	1	1	0	0	13

## Data Availability

Relevant data are within the manuscript and supporting information files. In this study, transcriptome data of the head, wings, and antennae had been submitted to NCBI (GenBank BioProject ID: PRJNA974177). Br-CA transcriptome data were downed from the Genome Sequence Archive in National Genomics Data Center under accession number CRA003038.

## Supplementary Materials

The following supporting information can be downloaded at https://www.mdpi.com/article/10.3390/genes14071427/s1. Table S1: comparison of the identity of TRP superfamily amino acid sequences between *L. migratoria* and other insects. Table S2: the accession number of TRP superfamily genes used in this study. Table S3: primers used for qRT-PCR assays in this study.
